# Design of Split Hexagonal Patch Array Shaped Nano-metaabsorber with Ultra-wideband Absorption for Visible and UV Spectrum Application

**DOI:** 10.1186/s11671-019-3231-4

**Published:** 2019-12-26

**Authors:** Ahasanul Hoque, Mohammad Tariqul Islam, Ali F. Almutairi, Mohammad Rashed Iqbal Faruque

**Affiliations:** 1 Department of Electrical, Electronic and Systems Engineering, Faculty of Engineering and Built Environment, Universiti Kebangsaan Malaysia, 43600 Bangi, Selangor Malaysia; 2 Electrical Engineering Department, Kuwait University, 13060 Kuwait City, Kuwait; 3 Space Science Center (ANGKASA), Universiti Kebangsaan Malaysia, 43600 UKM, Bangi, Selangor Malaysia

**Keywords:** Absorber, Dielectric property, Metamaterial, Photo-quantum, Solar energy

## Abstract

Solar energy is one of the ambient sources where energy can be scavenged easily without pollution. Intent scavenging by the solar cell to recollect energy requires a state-of-the-art technique to expedite energy absorption to electron flow for producing more electricity. Structures of the solar cell have been researched to improve absorption efficiency, though most of them can only efficiently absorb with narrow-angle tolerance and polarization sensitivity. So, there is a strong demand for broadband absorption with minimal polarization sensitivity absorber, which is required for effective solar energy harvesting. In this paper, we proposed a new Split Hexagonal Patch Array (SHPA) shape metamaterial absorber with Double-negative (DNG) characteristics, which will provide a wide absorption band with low polarization sensitivity for solar spectrum energy harvesting. The proposed new SHPA shape consists of six nano-arms with a single vertical split which with arrowhead symmetry. This arm will steer electromagnetic (EM) resonance to acquire absolute negative permittivity and permeability, ensuring DNG property. This DNG metamaterial features analyzed based on the photoconversion quantum method for maximum photon absorption. The symmetric characteristics of the proposed structure enable the absorber to show polarization insensitivity and wide incident angle absorption capabilities. Simulated SHPA shows a visible and ultraviolet (UV) spectrum electromagnetic wave absorption capacity of more than 95%. The quantum method gives an advantage in the conversion efficiency of the absorber, and the numerical analysis of the proposed SHPA structure provides absorbance quality for THz regime energy harvesting through solar cell or photonic application.

## Introduction

Material engineering has been contributing to the human history of development from ancient times, and ‘metamaterial’ is going to be one of the vital steering breakthroughs soon. ‘Meta’, denoting a change in the genre of material, shows unique dielectric characteristics like negative permittivity and permeability, easy to fabricate [1]. Different application potentiality [2, 3] in metamaterial makes several researchers around the globe more curious to do benchmark innovation in their respective research fields. Photonic energy conversion from visible frequency range and incorporate it in energy harvesting, specifically solar cell-based energy research, is one of the promising areas in metamaterial absorber [4–6]. Visible spectrum or UV range light waves surrounded us always without severe issues and an abundant amount of energy. Among all established techniques of utilization, photovoltaic (PV) technologies are widely applied to field application, and in the last few years, the state-of-the-art method has been proposed to improve performance to make the balance in future green energy challenges. For instance, single, multi-crystalline, and polycrystalline cells for efficiency improvement, PV development using metal halide perovskites, organic and quantum dot PV for power conversion efficiency enhancement, optoelectronic quality of PV relevant materials that affect the power output [7] and so on. Furthermore, material fabrication method like sequential deposition of high-quality PV perovskite layer [8], coated and printed PV perovskites [9], photon recycling [10] or algorithm based on centroid analogy at maximum power point [11], etc. are focused to enhance the efficiency of the solar cell.

Besides, a potential field of solar energy harvesting using a combination of antenna and rectifier (diode) known as ‘rectenna’ also been explored to enhance the efficiency of a typical PV cell. Rectennas have been studied mainly for microwave-based power transmission since it is highly efficient at converting microwave energy to electricity. For example, a prototype patented [12] using nanotechnology focused on converting light into electricity with enhanced efficiency and currently compatible with the traditional solar cell. The experimental procedure shows that the rectenna placed underneath a PV module gave an output 380 to 480 W/m^2^ with a combined module increased from 10–20% to 38–40%. Due to the nanofabrication technique constraint, most of the prototype operates in the far-infrared range rather than the visible spectrum. It can be expected that nanotechnology development may further expedite this approach. Thus, recent articles adopted a diverse strategy to harvest solar energy, such as the hybridization of RF-solar energy by the multiport transparent antenna [13] achieved 72.4% efficiency with 53.2% RF-to-DC conversion efficiency. Evolutive dipole nanoantenna (EDN) [14] fabricated by e-beam lithography dedicated to efficiency optimization for harvesting where efficiency increased from 30% to 40% compared to classic dipole nanoantenna (CDN). Metal-insulator-metal (MIM) integrated with SiO_2_ tunnel [15] shows conversion efficiency more than 90%, Zhang and Yi [16] proposed a similar approach using bow-tie shaped nano-rectenna claimed conversion efficiency of 73.38%. Likewise, metamaterial inspired rectenna with embedded Schottky diode-based ‘Fabry-Perot (FP)’ resonator [17] demonstrated high Q factor and 16 times performance improvement, optical rectenna inspired by metamaterial and developed by semi-classical model states high-efficiency, low-cost solar cell [18]. Not only that, several variations in metamaterial characteristics explored like switchable metamaterial with bifunctionality of absorption [19], vanadium dioxide-based thin metasurface, germanium inspired metasurface for tunable sensing [20]. Apart from the conventional idea of energy harvesting, most of the metamaterial absorber or antenna developed for RF energy harvesting rather than the visible spectrum. Energy harvesting in these articles [21, 22] unable to contribute to the solar cell.

Recent research in THz range rectenna or metamaterial absorber inspired nano-rectenna still under laboratory experiment or analysis because of several constraints like impedance matching, integration between unit cell and PV cell, feeding of converted energy from the unit cell to PV unit, photon conversion efficiency, transportation losses, etc. Moreover, PV cells likely to degrade performance with environmental parameters and narrow absorption band in the visible spectrum. Nevertheless, nanoscale antenna or absorber is being explored by adopting advanced design and fabrication technique such as omnidirectional structure plasmonic absorber [23] with harvesting efficiency about 38%, flexible substrate nantenna electromagnetic collector (NEC) [24] shows 90% absorption by overcoming optical behavior of materials and fabrication constraints. Unique optical and electrical properties of nanoscale structure [25–29] reveal a variety range of absorption percentages with dynamic material characteristics. Although most of the reported sophisticated structure yet challenging to apply in solar energy scavenging some metamaterial absorber used for intended application on an experimental basis [30, 31]. With the antenna converting the incident EM wave into an AC signal, the diode can rectify it to the usable DC voltage. Over 90% of conversion efficiency can be obtained in the radio frequencies. However, it is tremendously difficult to extend the rectenna to the optical regime due to the complicated process and the far too slow response of the diode-based rectification. A rarely noticed work on a direct photoelectric conversion without diode, known as dynamic Hall effect (DHE), was reported by H. Barlow in 1954. It was proposed to produce DC voltage via the joint action of dynamic electric and magnetic fields of the obliquely incident radiation. This effect is theoretically exhibited by all conducting materials and applicable to whole EM spectra from microwave to visible frequencies with a swift response [32]. Thus, a potential field of solar energy harvesting system efficiency enhancement using metamaterial yet to explore, analyze, and redeploy all available techniques to expedite typical solar cell efficiency at the application level.

**Fig. 1:**
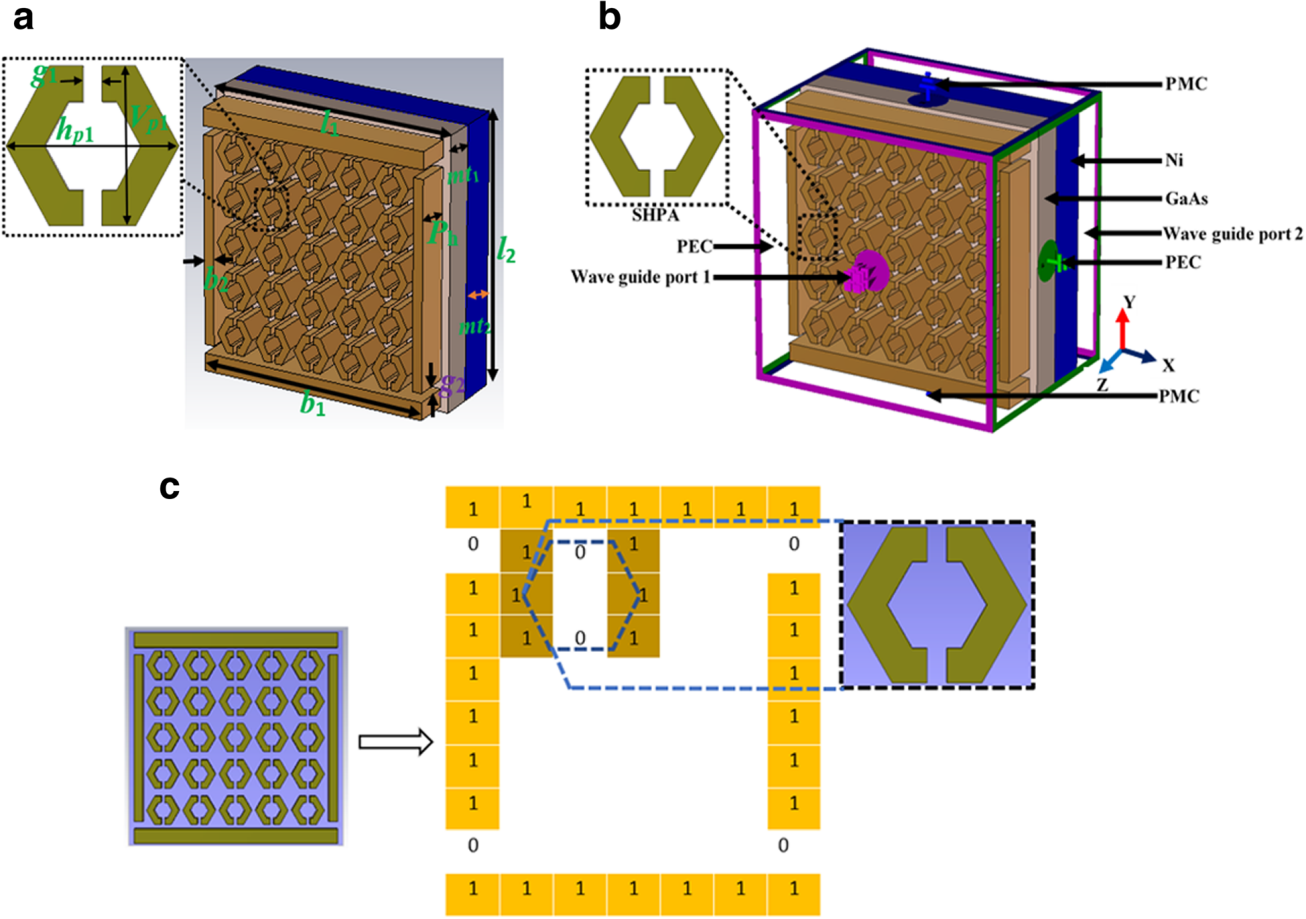
SHPA nano-metaabsorber. **a** Physical dimension. **b** Simulation set up. **c** GA-optimized encoding illustration

In this paper, we put forward an SHPA metamaterial absorber on tri-nanolayer material with DNG characteristics simulated both on visible and UV regime for solar energy harvesting. Finite-difference time-domain (FDTD) analytical method followed to structure formation, analysis, and commercially available CST Microwave Studio (MWS) 2017 used for simulation. Therefore, standard boundary conditions applied for wave propagation analysis as well as TE, TM plane polarization also modeled for wide-angle absorption. For structure optimized nano-range metamaterial absorber, genetic algorithms (GAs) have been successfully applied in many different designs to obtain a positive result [33, 34]. Hence, the proposed absorber adopted a similar algorithm [33] to find the negative index material (NIM) characteristics. Figure 1c illustrates GA-optimized unit cell design domain where nano split Hexa shape and divided 10 × 7 grid. Inside the grid, a subdivided 3 × 3 grid depicts hexagonal shape. The actual mechanism is interpolation of data to get the improved absorption varying geometrical dimension while preserving nanostructure shape. The goal of this GA is to extract SHPA metamaterial for visible frequency with maximum possible NIM characteristics. Scattering parameter evaluated during simulation procced to MATLAB program to extract characterization and relevant property analysis. Numerical investigation shows more than 95% absorption in both frequency regime with significant left-handed metamaterial characteristics. Thus, proposed SHPA with further fabricated validation can prove its potential application field like solar energy harvesting, photon accumulation process for a solar cell, or photonic amplification.

## Computational Design and Methodology

SHPA metamaterial absorber was modeled as a double-layer substrate, Gallium arsenide (GaAs), and Nickle (optical), and patch layer designed on Gold (Au). An 80-nm-thick GaAs with lossy permittivity of 12.94 and 100-nm-thick Ni (Fig. 1a). Table 1 shows the detail dimension of the unit cell structure. The thickness of the SHPA patch is 90 nm, and Au film is negligible to a localized magnetic field, isotropic conductivity of 4.1 × 10^7^ S/m [35]. According to ‘anisotropic Drude conductivity tensor’ [36], only the Z-component of the local magnetic field considered. Because an orthogonal component of the other two axes is much weaker than the Z-component. During simulation periodic boundary condition in X and Y direction applying PEC (perfect electric conductor) and PMC (perfect magnetic conductor) respectively on the top and bottom layer (Fig. 1b). Anisotropic conductivity on unit cells was ensured by incorporating a localized magnetic field. The S-parameters of SHPA were simulated, ranging from 430 THz to 1000 THz with the step size of 100 THz. The reflection (R), transmission (T), and absorption (A) range obtained by A =1-T-R where |*S*_11_|^2^ = R and |*S*_21_|^2^ = T. Plane-wave of electric field defined by*E* = *E*_*x*_Cos(*ωt* + *kz*) propagating towards the Z-axis where *E*_*x*_ is the amplitude of the electric field, ω is angular frequency, *t* is time, and *k* is wave number.

**Table 1:** Proposed SHPA unit cell dimension

Substrate and HEXA patch (nm)							Patch bar dimension (nm)		
*l*_1_ and *l*_2_	*g*_1_	*V*_p1_	*h*_p1_	*P*_h_	mt_1_	mt_2_	*b*_1_	*b*_2_	*g*_2_
600	10	77.94	90	90	80	100	561	39	20

Geometrical structure development for metamaterial suggested by Pendry [37] widely applicable for microwave range but THz regime, i.e., visible and optical frequency shows major disadvantages in negative permeability and parallel propagation multilayer substrate. So, an alternative design approach [38] metal-dielectric-metal illustrate good response as a resonant magnetic dipole for normal propagation to the structure which demonstrates negative permeability and simplified layer structure is relatively easy to fabricate in nanoscale. Moreover, designing metamaterial absorber with DNG properties in three dimensional requires several characteristics on the structure such as backward propagation, reversed Doppler effect, evanescent wave amplification, etc. Although theoretical analysis and capabilities regarding the visible frequency spectrum already been described by the experts [39–41]. Thus, the thin-film nanostructure DNG characteristics-based MA is concerned with negative *ε* and *μ* and commonly employed as a periodic thin metallic array. Thin metallic patch array dilutes free-electron plasma described by ‘Drude’ model but as we have considered upper layer as lossy hence

1 $$ \varepsilon ={\varepsilon}_0{\varepsilon}_r\left(1-\frac{{\omega_p}^2}{\omega^2}\right)\;\mathrm{and}\;\mu ={\mu}_0{\mu}_r\left(1-\frac{M_m^2}{\omega^2-{\omega}_m^2+ j\omega {\gamma}_m}\right) $$

where *ω*_*p*_ is reduced plasma frequency depends on the geometrical dimension of a thin layer, *ω*_*m*_ is magnetic resonance frequency, *γ*_*m*_ losses, *M*_*m*_ determines the strength of the magnetic resonance.

## Results, Analysis, and Discussion

### Unit Cell Power and Dielectric Properties

According to the photo-quantum method, a certain amount of power requires at the boundary condition of the unit cell, especially in the propagation direction, polarization angle, E-field and H-field current flow, etc. So, let us analyze the power which is required to propagate in a multi-crystalline direction [42]. Equations (2) and (3) are based on a complex Poynting vector theorem inspired by [42, 43]. The fact is power receiving by the unit cell would be sunlight, which is omnidirectional, and power flow using the absorber must go in a direction to enhance the efficiency. Thus, the power of the propagating wave is just proportional to the real part of the vector related to the time-average parameter. Stimulated power at one or both ports will propagate through the unit cell. The rest of the energy will leave through all ports (outgoing power). Accepted power in the unit cell is converted in losses like dielectric materials properties, patches, or lumped elements considered for SHPA nano-arms. Considering the real part of complex average power in *Z*-direction

2 $$ {P}_{c\left(\mathrm{avg}.\right)}=\operatorname{Re}\left\{\frac{1}{2}\underset{A}{\int}\overrightarrow{E}\times \overrightarrow{H}.\mathrm{zdz}\right\} $$

Which is also valid for (Z_-ve_ direction) to describe the net flow of energy at a specific port. The ½ factor in Eq. (2) is related to time-averaging the clockwise field. The imaginary part of the power can be ignored due to non-propagating reactive or stored energy and can calculate the transmitted power (P_T_) observing the average time power along *X* and *Y* axis respectively-

3 $$ {P}_{T\left(\mathrm{avg}.\right)}=\frac{\operatorname{Re}\frac{1}{2}\underset{A}{\int }{P}_y.\mathrm{dy}}{\operatorname{Re}\frac{1}{2}\underset{A}{\int }{P}_x.\mathrm{dx}} $$

**Fig. 2:**
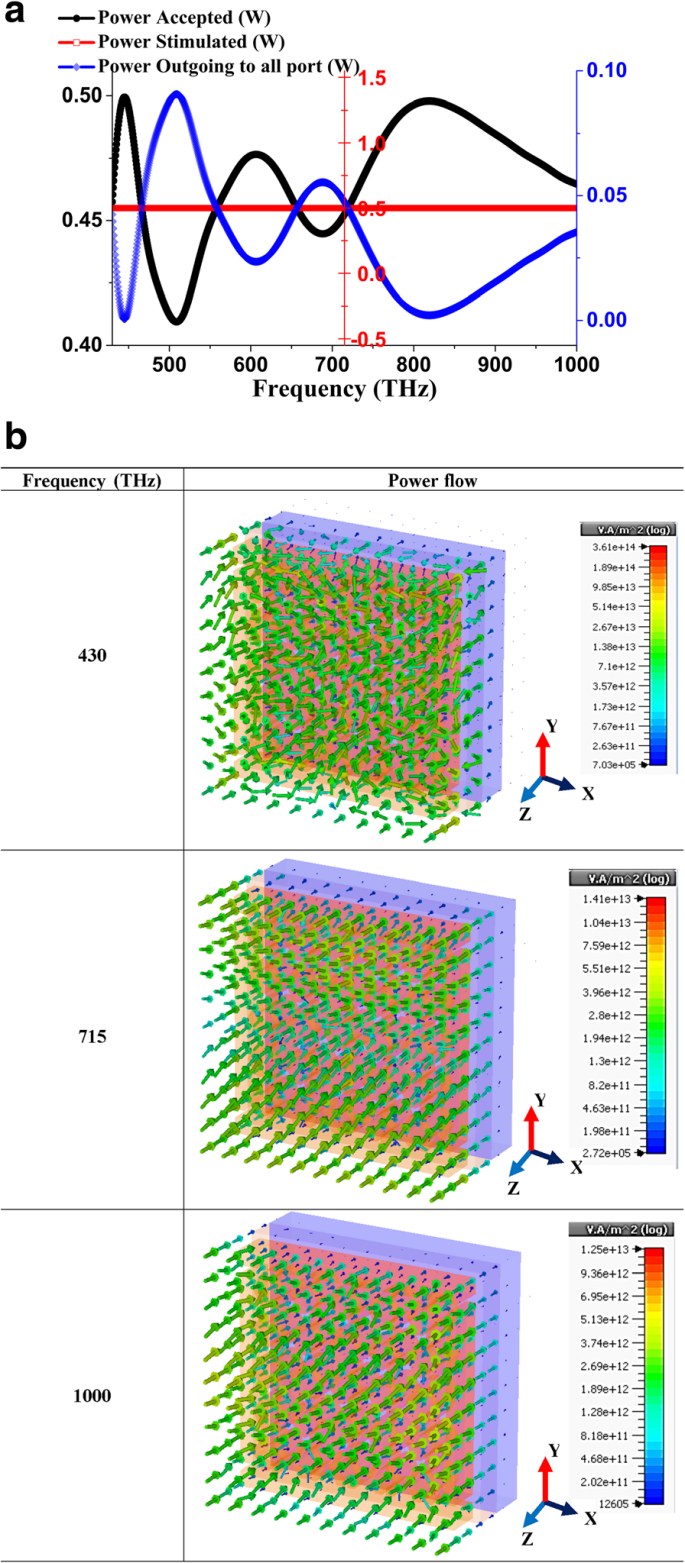
Power distribution in SHPA metaabsorber (**a**) 2D distribution (**b**) 3D power flow through the unit cell

Similarly, accepted and outgoing power was calculated using the equation in [43] and plotted in Fig. 2 where associated power (Fig. 2a) and power through the unit cell (Fig. 2b) nano-metaabsorber observed during the simulation. Stimulated power limited to 0.5 watts in the whole spectrum, while accepted and outbound power in both ports has vice versa power distribution. However, 3D power flow shows unusual characteristics due to dipole moment inertia with the operating frequency range and nonhomogeneous material penetration state. Starting from 430 THz, most of the dipole moment is not organized since THz operation at the initial stage has polarization effect and steadily having proper dipole effect aft 715 THz, which continued up to 1000 THz. Besides, GaAs material semiconductor property, as well as Ni’s ferromagnetic characteristics, are responsible for deterring the

power flow but fortunately not so dominating. Dielectric properties (*ε*, *μ*, *η*) extracted from S-parameter for the numerical investigation to assess metamaterial properties. The unit cell absorber with three different materials have isolated characteristics in EM wave propagation, but this unique structural dimension with cascaded capacitance and inductance on top patches modify the conventional properties of individual material dielectric features and depicts unique properties. Now, extracting the dielectric properties DRI method [44] used where transmission coefficient (S_21_) and reflection coefficient (S_11_) was the critical parameter.

**Fig. 3:**
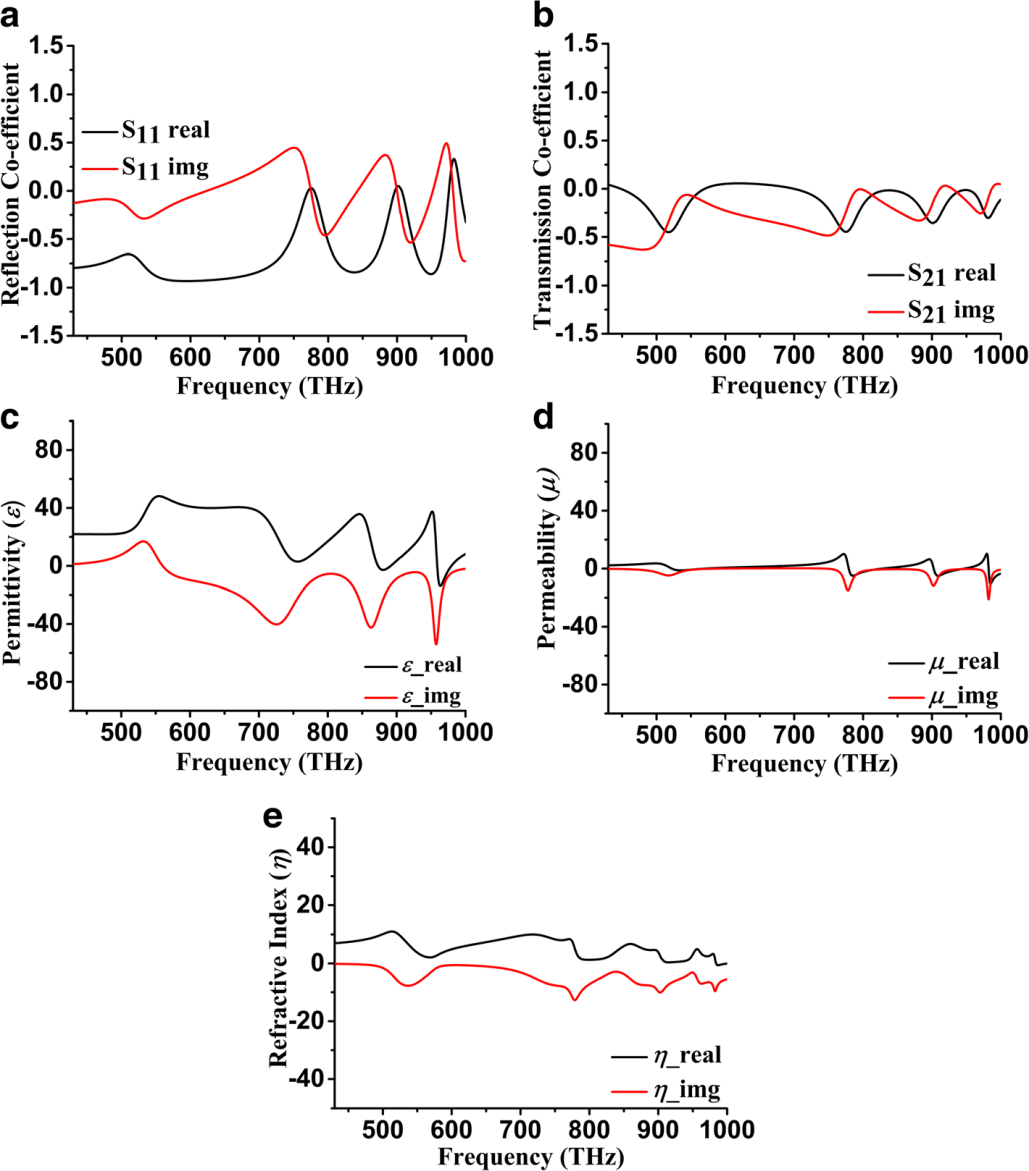
SHPA metamaterial characteristics. **a** S_11_ Response. **b** S_21_ Response. **c** Permittivity. **d** Permeability. **e** Refractive index over visible and infrared spectrum

**Fig. 4:**
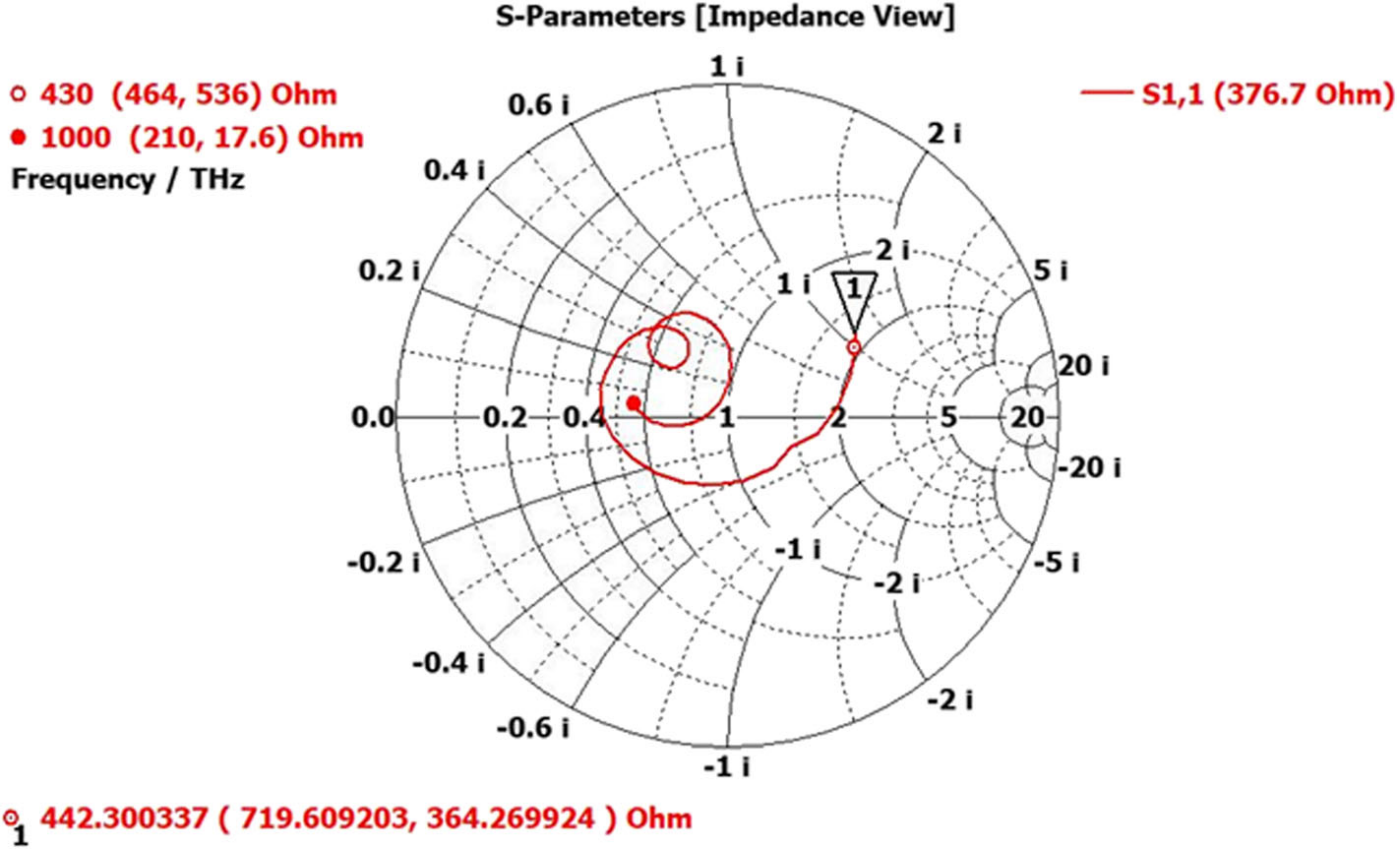
Smith chart shows VSWR of unit cell SHPA over the spectrum at a normalized impedance

Figure 3 shows all the simulated results of the proposed SHPA nano-metaabsorber. Figure 3a,b magnitude of S_11_ and S_21_ has almost consistent magnitude both in the real and imaginary part. Although infrared range response has three consecutive small resonance points because of skin depth (δ) effect of structure, fortunately, it plays a positive role in getting the negative permittivity, permeability, and refractive index. Figure 3c,d,e respectively shows the real and imaginary value of these properties and ensure the metamaterial existence on proposed SHPA. Furthermore, intense thermal electromagnetic evanescent fields [45] are needed to be considered due to the application perspective of solar energy harvesting. Experimentally mentioned in [45, 46] that, during near-field radiation, two consecutive material heat conduction gradually increase. Besides, surface polaritons also dominate the evanescent waves and according to the ‘Drude model,’ complex permittivity and permeability determined by wave polarizations inside the unit cell. Figure 3 c,d,e presents dielectric properties where lower wavelength operation of permittivity and permeability affected by this evanescent wave. Hence, the negative characteristics of the proposed unit cell significantly visible and ensure good EM absorption. Transmission line characteristics and VSWR (voltage standing wave ratio) of the SHPA nano-absorber in Fig. 4 clearly show reflection amount

and transmission line performance. VSWR at 430 THz impedance was high, and half-wavelength of the line does not have a good matching from source to load side. Hence, the EM signal absorption amount is also low at a lower frequency, but gradually, the impedance tried to match (with normalized one) as much as possible resultant with above 90% absorption at infrared spectrum (1000 THz). As the unit cell represents an absorbing element rather than a radiating element; hence, its VSWR at the load side does not have a higher value.

### Field-Effect Analysis

**Fig. 5:**
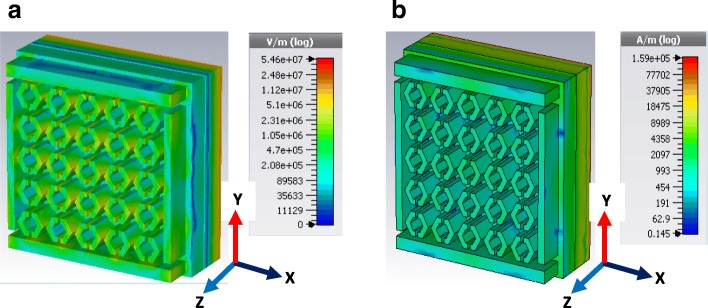
Field effect on SHPA at resonance 550 THz. **a** E-field. **b** H-field

EM nature of light is a transverse electromagnetic wave at the visible regions. The light coming from the sun is divided into three spectra: infrared, visible, and ultraviolet (UV). Spectral energy distribution of solar light has maximum intensity 1.5 eV at a visible range similar to most semiconductor material while two other spectra produce heat if absorbed. So, considering typically visible light EM propagation and boundary conditions stated in Fig. 1b, the electric field (E-field) and magnetic field (H-field) numerical performance is shown in Fig. 4. Though resonance frequency 445 THz characteristics present in the figure but whole bandwidth 430~650 THz have a similar distribution of the field. Now, vector wave equations as mentioned in [47]

4 $$ {\displaystyle \begin{array}{l}{\nabla}^2{E}_m-{\gamma}^2{E}_m=0\\ {}{\nabla}^2{H}_m-{\gamma}^2{H}_m=0\end{array}}\Big\} $$

where one-dimensional vector differential operator∇varies slightly with phase variation during EM wave propagation, electric, and magnetic field components are*E*_*m*_and *H*_*m*_ respectively, propagation constant $\gamma =\sqrt{j\omega \mu \left(\sigma + j\omega \varepsilon \right)}$is a complex quantity related to attenuation and phase deviation of the wave. Since the visible light wave has both wave and particle property, wave propagation through the unit cell material shows variation in terms of E-field and H-field characteristics. Furthermore, *γ* have a non-linear relationship with dielectric properties as operating frequency gradually increases. Figure 5 shows each nano split on SHPA significant E-field component (2.31 × 10^6^ V/m in log scale) exist at resonance 550 THz. Though over the simulated frequency region (visible and UV), this strong E-field observed with slight variation in amplitude. Horizontal and vertical patch bar (with four splits) also contribute field component with amplitude variation (2.08 × 10^5^~2.31 × 10^6^ V/m log scale). During a transient analysis of SHPA unit cell (two-stage cascade) given the capacitance and inductance value of 1.37 × 10^−17^ nF and 3.87 × 10^−14^ nH accelerate the resonance frequency field operation. H-field (Fig. 5b) has a similar effect from EM propagation along Z-direction, and during inhomogeneous medium penetration, Eq. (5) becomes functions of *Z* and in which the magnetic permeability constant. Then the corresponding wave equation is reduced to a “Ricatti differential equation” [48]

5 $$ \frac{d\psi (z)}{d z}+{\psi}^2(z)=-{k}^2{m}^2(z) $$

where *k* is wave number, and *m(z)* is a complex refractive index. Furthermore, phase retardation of the wave increases with the ratio of phase velocity in free space and medium, which is another significant contribution of the proposed unit cell SHPA for lower reflectance and absorbs more energy from the wave.

**Fig. 6:**
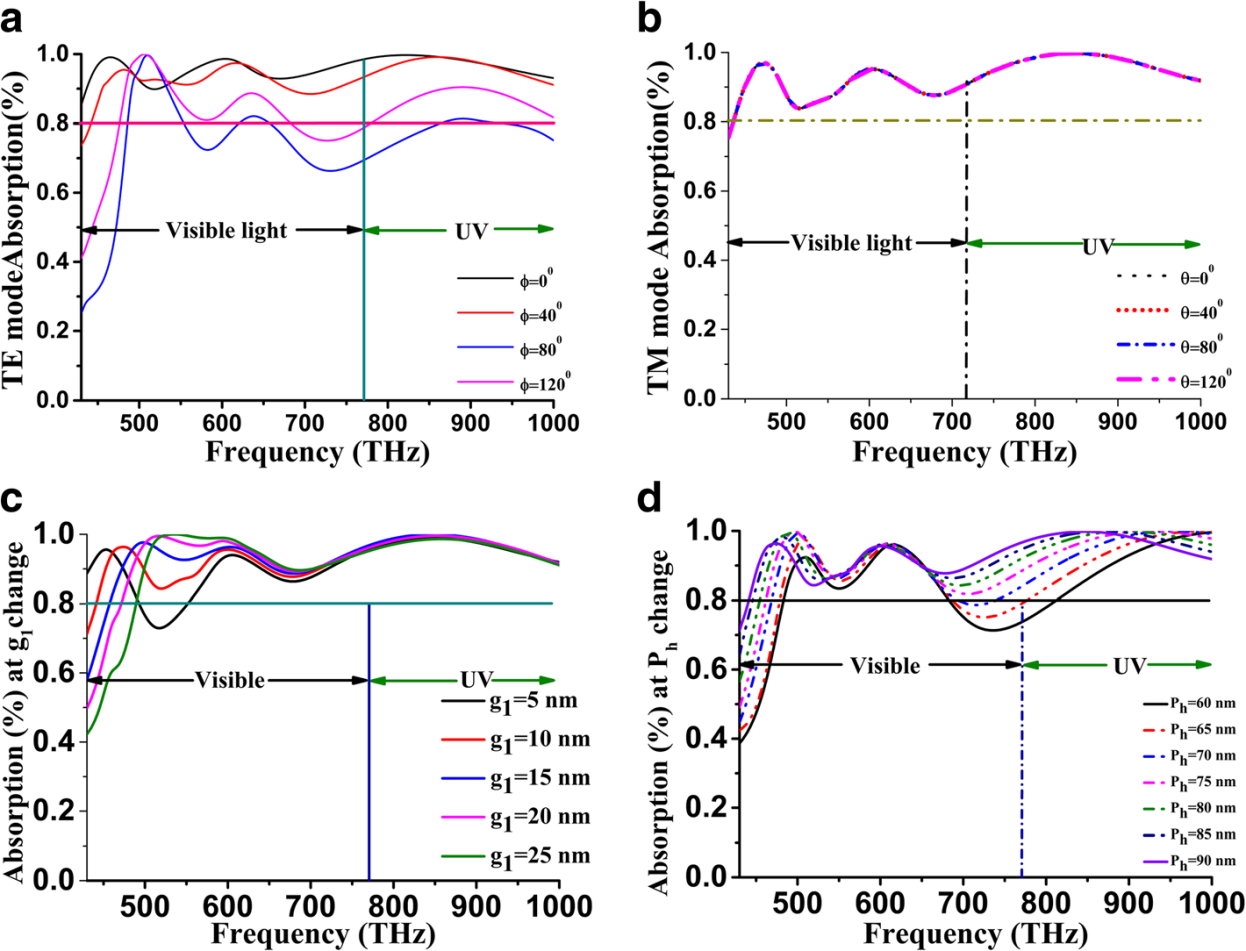
Polarization effect on absorption. **a** TE polarization. **b** TM polarization and SHPA structure effect. **c** Split gap vs. absorption. **d** Height vs. absorption

Polarization of lightwave studied on proposed unit cell SHPA to explain unit cell feasibility for solar energy harvesting since the polarized wave through the surface loses its energy during propagation. Hamiltonian formulation [49] mentioned that transition dipole matrix elements vary for TE and TM polarization in the different incident angles of the wave on GaAs material. The polarization angle both for TE and TM mode increase step size of 40 ° (Fig. 6), and the electric field polarization angle has a surprisingly dominating effect compare to magnetic field orientation. During TE mode, at a lower range, approximately 430–650 THz (690 nm to 460 nm) [50], for a given difference of Ni-GaAs substrate combination, the difference between core and cladding layers makes a varying refractive index which increases when visible wavelength approaches the bandgap. Hence, the fluctuation of absorption amount observed on that spectrum (Fig. 6a), whereas TM polarization shows similar type fluctuation despite polarization angle changes from 0 ° to 120 °. At TM mode, phase mismatch generally becomes large for longer wavelengths. Besides, the hexagonal shape has a significant effect on absorption during variation of split gap and height of the patch. Capacitance formed by the split gap patch is varying whereas the adjacent capacitance by the position of the patch is stand. Figure 6c split gap change from 5 nm to 25 nm and lower the split gap give excellent absorption because of substantial capacitance. Despite gap change, absorption nearly remains above 90% for 5 nm, and the gradual increase of split gap makes an initial absorption drop around 430–500 THz but overall 95% absorption observed during the simulation. In terms of SHPA height (Fig. 6d), as the patch split remains 10 nm, the EM signal propagation area collectively increases both for normal and oblique incidence and hence split height optimized with higher value with absorption. For SHPA height or thickness 60 nm to 90 nm average absorption 85% to 88%, which directly states the optimized for 90 nm.

However, a fabricated prototype and measure results of SHPA would support simulated data, which will be carried on the next phase of the study. Besides, a comparative picture described in Table 2 to understand the contribution of proposed nano-metaabsorber. In Table 2, the reported article [51] shows good efficiency, but the operating frequency and narrowband performance make it unable to comply with visible frequency operation. Another article [52, 53] claimed for solar energy harvesting applications, but bandwidth and operating range make it more vulnerable compared to others.

**Table 2:** Comparative study of related metamaterial absorber for solar energy harvesting

Paper	Dimension (nm)	Absorption (%)	Operating band (THz)	Material name	Application	Remarks
[51]	1600 × 2600 × 220	41	300–420	InAs/GaInAsSb	Thermophotovoltaic	Narrowband
[52]	550 × 610 × 200	86.35	200–1000	Pyrex/dielectric/Au	Solar energy harvesting	Narrowband
[53]	400 × 400 × 200	91	700–1000	Au/Si multilayer	Solar energy harvesting	Ultra-broadband
Proposed	600 × 600 × 90	95	430–1000	Au/GaAs/Ni	Solar energy harvesting	Ultra-broadband

## Conclusions

In this paper, a split hexagonal metamaterial absorber is proposed using Au six nano-arms based on GaAs and Ni substrate for solar energy harvesting applications. Photo-quantum analysis and power flow distribution mathematically show that the proposed unit cell has significant photon conversion possibility for photovoltaic or solar cell applications. The performance of proposed unit cell SHPA was analyzed based on dielectric properties, transmission line performance, field and power distribution, absorption in terms of the parametric study. All the data were extracted from S-parameter through CST MWS simulation, which shows that DNG characteristics exist with ultrawideband EM absorption (more than 95%) both in the visible and UV spectrum of light. Optimized Hexa patch unit is a 10 nm split gap and height of 90 nm for stated absorption. Experimental validation of the proposed absorber will be further continued to be a desirable candidate in THz range energy harvesting applications.

## Data Availability

All data are fully available without restriction.

## Abbreviations

CDN: Classic dipole nanoantenna
DRI: Direct refractive index
DNG: Double negative
EM: Electromagnetic
FDTD: Finite-difference time-domain
GA: Genetic algorithm
PV: Photovoltaic
SHPA: Split hexagonal patch array
UV: Ultraviolet
